# The impact of the COVID-19 pandemic lockdown on rhegmatogenous retinal detachment services—Experiences from the Tongren eye center in Beijing

**DOI:** 10.1371/journal.pone.0254751

**Published:** 2021-08-19

**Authors:** Jipeng Li, Meng Zhao, Haicheng She, Aman Chandra

**Affiliations:** 1 Department of Ophthalmology, Beijing Tongren Eye Center, Beijing Key Laboratory of Ophthalmology and Visual Science, Beijing Tongren Hospital, Capital Medical University, Beijing, China; 2 Department of Ophthalmology, Mid & South Essex NHS Foundation Trust, Southend University Hospital, Anglia Ruskin University, Cambridge, United Kingdom; Massachusetts Eye & Ear Infirmary, Harvard Medical School, UNITED STATES

## Abstract

**Purpose:**

To investigate the impact on services for rhegmatogenous retinal detachment (RRD) patients during the COVID-19 (2019coronal virus disease) pandemic in one tertiary center in Beijing.

**Methods:**

A retrospective cohort study. Two reviewed consecutive RRD patients cohorts of the same length were treated during two different periods: the COVID-19 pandemic and the pre-COVID-19 group. The characteristics of patients, surgery, anesthesia methods, length of hospital stay, and the latest follow-up were recorded and analyzed.

**Results:**

There were 79 patients in the COVID-19 pandemic group with a 55.9% reduction (179). Compared to patients in the pre-COVID-19, patients in the COVID-19 pandemic had a longer presurgical waiting times (28days, 3days, p<0.001), a higher percentage of patients with presurgical poor (less than 0.02) visual acuity (55.7%, 32.4%, p = 0.009), and a higher percentage of patients with presurgical choroidal detachment (34.2%, 19.6%, p = 0.01). There was no significant difference in the severity of presurgical proliferative vitreoretinopathy between the two groups (p = 0.64). Surgeries on pathological myopia patients with macular hole retinal detachment were postponed in the COVID-19 pandemic. There was a lower percentage of scleral buckling (27.8%, 41.3%, p = 0.02) and a lower rate of subretinal fluid drainage (45.4%, 75.7%, p = 0.01) in the COVID-19 pandemic. There was no significant difference in either postoperative visual acuity (p = 0.73) or the rate of single-surgery retinal attachment (p = 1) between the two groups. Patients in the COVID-19 pandemic had a shorter length of hospital stay (3hours, 35 hours, p<0.001), and a lower percentage of patients received general anesthesia (48.1%, 83.2%, p<0.001). None was infected with COVID-19 disease during the pandemic.

**Conclusion:**

The COVID-19 pandemic lockdown caused prolonged presurgical waiting times, shorter hospital stays, less general anesthesia, and a significant reduction of RRD surgeries. The RD were more complicated, the surgeons were more conservative on procedures and patients selection, while the surgery outcomes were comparable.

## Backgrounds

In December 2019, the global pandemic involving the highly contagious coronavirus disease 2019 (COVID-19) began. Beijing’s health authorities upgraded its emergency response to the top-level within three days after ten COVID-19 cases were confirmed on January 24, 2020. The Top-level response ended on April 29, 2020. In response to the pandemic, hospitals postponed most elective surgeries [1–3]. A 14-day quarantine period was required for all the admitted patients. During the closed-off management, local patients from high-risk communities were not allowed to leave their communities except for life-threatening emergencies.

Rhegmatogenous retinal detachment (RRD) is a sight-threatening ocular disease often requiring urgent surgical intervention [2]. It can lead to irreversible visual damage if it is not treated in time [4]. RRD patients experiencing long delays for surgery are considered more likely to undergo a second surgery within 30 days of the primary procedure [5].

Because the majority of in-patients followed the 14-day quarantine, an inevitable delay in treatment of RRD occurred. Meanwhile, the COVID-19 outbreak had an enormous impact on hospitalization, anesthesia, healthcare worker personal protective equipment, and outpatient service. In response to the pandemic, adjustments were proposed to minimize nosocomial spread in Beijing Tongren Hospital. These included 1) creating teams of ophthalmologists to deal with out-patients clinics and the in-patients surgeries separately (with one team operating in one negative-pressure operating room for a whole day); 2) requiring all the patients who needed surgeries to undergo the COVID-19 screening according to the latest protocol of prevention and treatment of the COVID-19 disease published by the Chinese government; permitting only the patients with a negative SAR-Cov-2 screening result to be treated in the operating center; 3) encouraging the sedation and retrobulbar nerve block over general anesthesia, wherever possible; 4) encouraging day-care service to reduce hospital stays; 5) minimizing postoperative follow-up visits in the outpatients’ clinics; 6) creating an isolation ward in case of admission of COVID-19 suspect patients. Although there were reports on other emergency surgeries performed in the same period [6, 7], and two reports on RRD’s characteristics during the COVID-19 pandemic [8, 9], no large-scale report on the treatment and surgery outcome of RRD during the pandemic has been reported yet.

The Beijing Tongren Eye Center was the only center that provided RRD surgery service in Beijing during the period of the COVID-19 pandemic. Herein we report our experience of RRD patients treated in the eye center during the COVID-19 outbreak compared with RRD patients treated before the COVID-19 pandemic.

## Methods

A consecutive cohort of RRD patients presented during the first 74 days of top-level response (February 16, 2020 –April 30, 2020) was compared to a consecutive cohort of RRD patients presenting in a pre-COVID-19 pandemic with the same length. The study followed the tenets of the Declaration of Helsinki, and the institutional review board of Beijing Tongren Hospital approved the protocol.

The following preoperative characteristics were collected: age, gender, presurgical waiting times (including the time from symptoms to the presentation and time from presentation to surgery), the history of previous eye trauma, surgical intervention details, presurgical visual acuity (VA), intraocular pressures (IOP), location of retinal breaks, phakic status, co-existence of pathological myopia (PM), the extent of retinal detachment (RD), concomitant proliferative vitreoretinopathy (PVR), choroidal detachment (CD) and co-existence of congenital vitreoretinal diseases.

All patients underwent either 23 G or 25G pars plana vitrectomy (PPV) or scleral buckling (SB) under local or general anesthesia. The following details were also collected: anesthesia method, type of surgery, and length of hospital stay. In SB cases, the segmental or radial buckle and the combination of encircling band’s placement and subretinal fluid drainage were recorded. In PPV cases, the PVR details were confirmed during PPV, the combination with SB or cataract extraction and the type of tamponade was recorded.

All patients were followed up on for a minimum of 3 months. CD was defined as presenting with CD, having the patient examined by using either indirect ophthalmoscopy or a B scan ultrasound [10]. PM was defined as myopia with combined changes of pathological characters [11].

Statistical analysis was performed using version 3.20 of R (http://www.R-project.org). Patient characteristics were retrieved from their medical charts and recorded in version2.0.3.15 of Epidata Entry Client (http://epidata.dk). VA results were converted to logMAR values for statistical analysis. Mean and standard deviation (SD) values were calculated for continuous variables with a normal distribution. Median with quartiles values were calculated for continuous variables with a non-normal distribution. The t-test or Mann-Whitney U test was carried out for continuous variables. The Chi-square test or Fisher’s exact test was carried out for discrete data.

## Result

There were 79 cases in the COVID-19 pandemic group, with a 55.9% reduction from the pre-COVID-19 period (179).

### Influence on RRD services

The median time from the onset of visual symptoms to surgery in the COVID-19 pandemic group was 28 days (7–366) and was much longer in the pre-COVID-19 group (4 days, 1–240, p<0.001). Seventy cases (88.6%) failed to present to the hospital within one week after the onset of visual symptoms.

Compared to the pre-COVID-19 group, the median length of hospital stay was shorter (3 hours, 2–288 hours vs. 35 hours, 24–456 hours, p <0.001), and the percentage of patients under general anesthesia was lower (48.1%, 83.2%, p <0.001) in the COVID-19 pandemic group.

### Influence on patients

**Fig 1:**
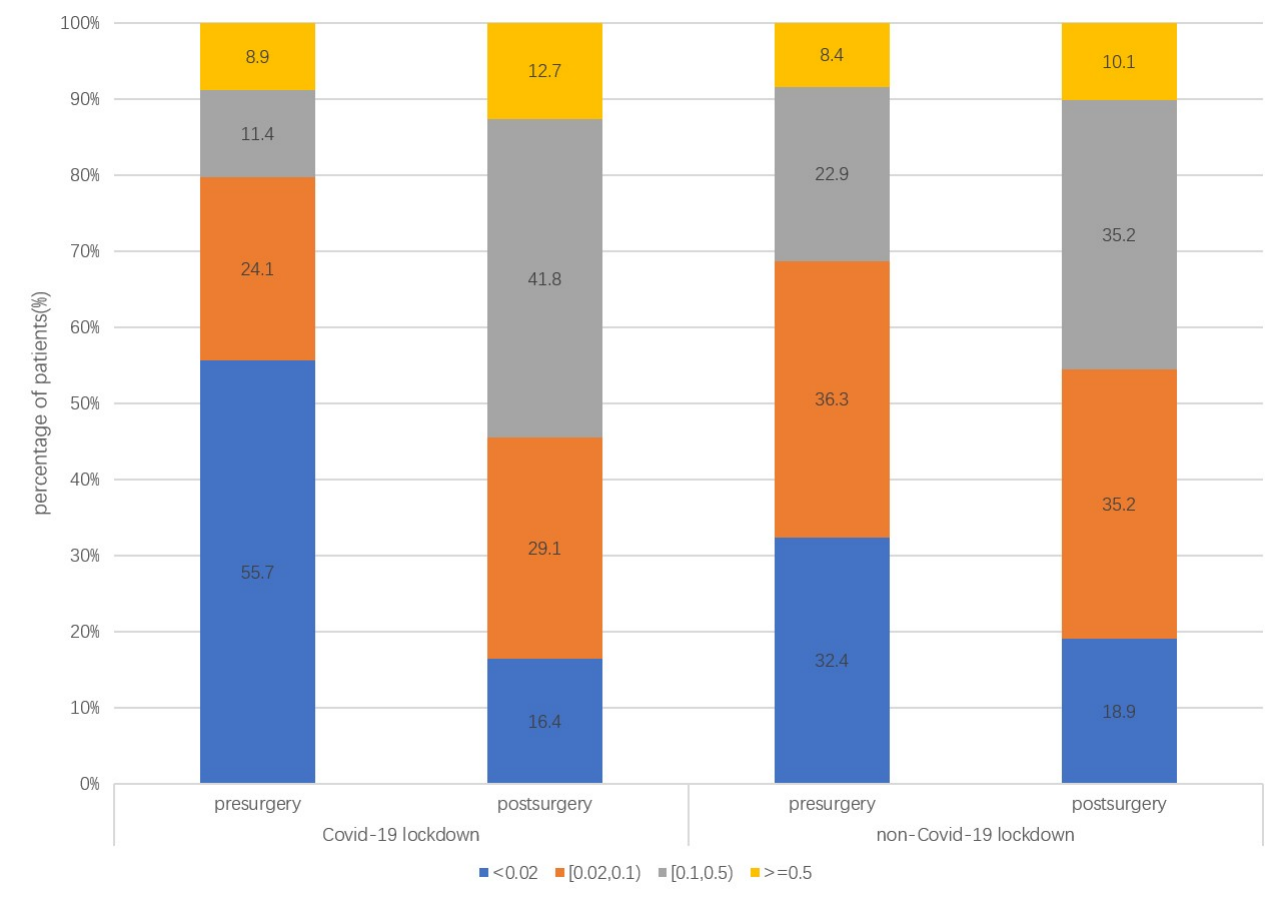
VA distribution of the patients. There were more patients with poor VA in the presurgical VA (less than 0.02) in the COVID-19 pandemic group than in the pre-COVID-19 group (55.7%, 32.4%, p = 0.009). There was no significant difference in the distribution of postsurgery VA in the two groups (p = 0.73).

Compared to the pre-COVID-19 group, there were more patients whose presurgical VA was less than 0.02 (55.7%, 32.4%, p = 0.009; Fig 1), more patients with RRD-CD (34.2%, 19.6%, p = 0.01), and more patients with pseudophakic eyes (22.8%, 13.4%, p = 0.047) in the COVID-19 pandemic group.

The percentages for PVR-B, PVR-C, PVR-D, and a combination with anterior PVR in the COVID-19 pandemic group (62.0%, 27.8%, 5.1%, 5.1%) were similar to those in the pre-COVID-19 group (61.5%, 24.0%, 5.0%, 9.5%, p = 0.64). The percentage of patients with four quadrants of RRD (53.2%, 43.6%, p = 0.14) or macular detachment (86.1%, 78.8%, p = 0.23) was comparable in the two groups.

In patients with RRD-CD, the location of the retinal breaks (p = 0.04), not the type (p = 0.66), was different between the two groups. There was a higher percentage of patients with retinal breaks posterior to the equator in the COVID-19 pandemic group than the pre-COVID-19 group (63.0%, 31.4%, p = 0.04).

There was no PM patient with macular hole RD in the COVID-19 pandemic group but there were 20 patients in the pre-COVID-19 group.

### Influence on the surgery (Table 1)

Compared to the pre-COVID-19 group, fewer patients received SB (27.8%, 41.3%) while more patients received PPV (72.2%, 58.6%, p = 0.02). In patients who received SB, fewer patients received subretinal fluid drainage in SB surgery in SB surgery (45.4%,75.7%, p = 0.01) in the COVID-19 pandemic group (Table 1). In patients who received PPV, fewer patients received PPV combined with cataract extraction (7.0%, 21.0%, p = 0.02) in the COVID-19 pandemic group. The prevalence of silicone oil (91.2%, 86.7%) or gas (8.8%, 13.3%) tamponade in patients was similar between the two groups (p = 0.45).

**Table 1:** The difference in treatment patterns between the two groups.

(n, %)	The COVID-19 pandemic group	The pre-COVID-19 group	P
PPV (n, %)	57, 72.2%	105, 58.6%	0.04
Combined with SB	1, 1.8%	0, 0%	
Combined with PHACO	4, 7.0%	22, 21.0%	0.02
Silicone oil tamponade	52, 91.2%	91, 86.7%	0.45
C3F8 tamponade	5, 8.8%	14, 13.3%	
SB (n, %)	22, 27.8%	74, 41.3%	0.01
Segmental buckle	8	15	0.29
Radial buckle	1	6	
Combined encircling	13, 59.1%	53, 71.6%	
drainage of subretinal fluid	10, 45.4%	56, 75.7%	0.01

### Influence on the surgery outcomes

The median of follow-up was 99 (61–152) days in the COVID-19 pandemic group and 216 (26–305) days in the pre-COVID-19 group.

**Fig 2:**
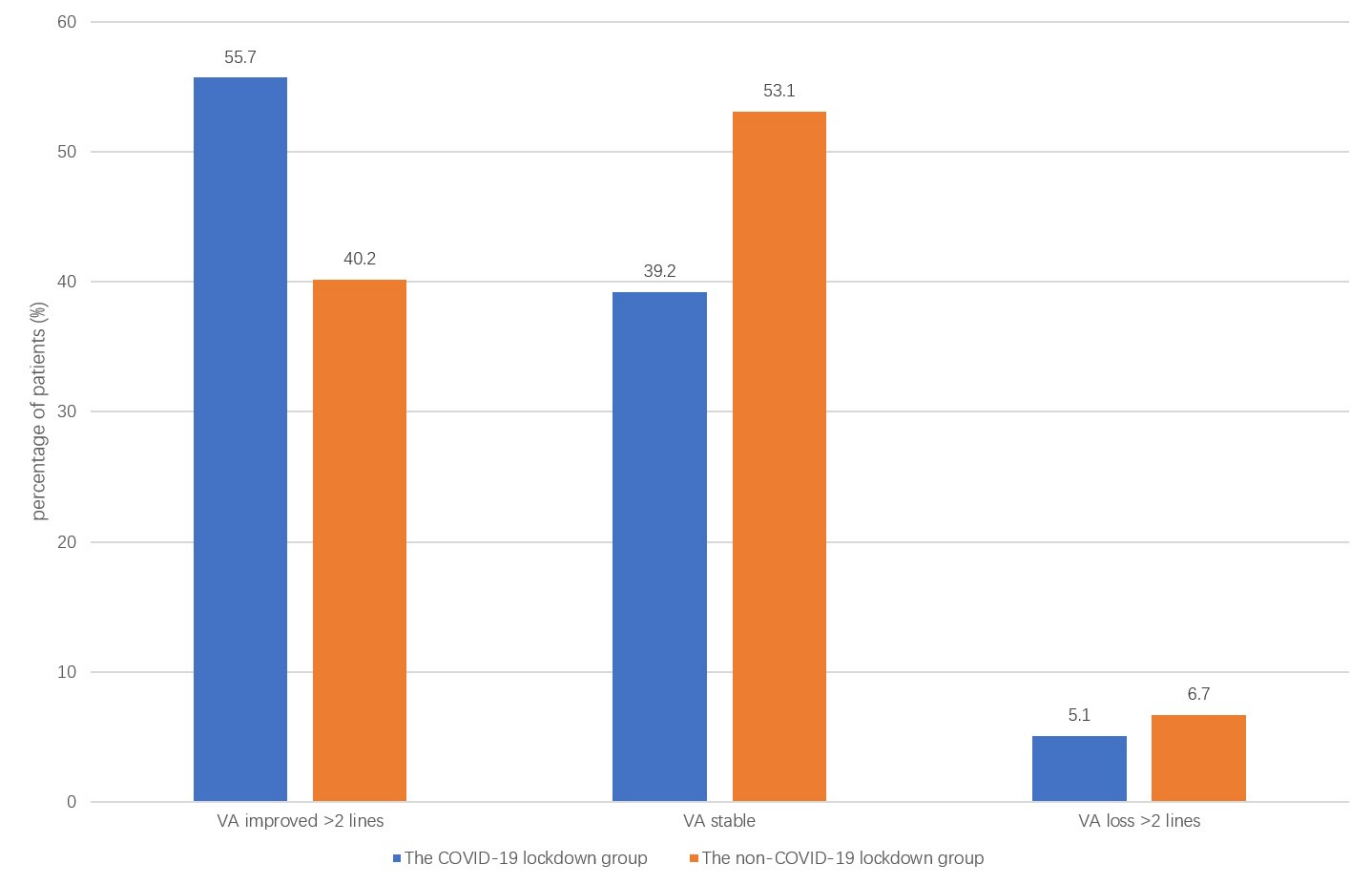
VA changes in the two groups. The percentage of patients with VA gained more than two lines, VA stable, and VA dropped more than two lines was similar between the two groups. There was also no significant difference in the final VA changes between the two groups (p = 0.08).

There was no significant difference in the final VA between the two groups (p = 0.73). There was also no significant difference in the final VA changes between the two groups (p = 0.08) (Fig 2).

The overall single-surgery anatomic retinal reattachment rate (SSRA) was similar between the two groups (94.9%, 94.5%, p = 1), and was consistent in those who received PPV (96.5%, 94.3%, p = 0.80) or SB (90.9%, 94.6%, p = 0.61). Ten patients in the COVID-19 pandemic group and 67 patients in the pre-COVID group had the record of silicone oil removal at our center before the latest follow-up.

The percentage of patients with postoperative IOP greater than 30mmHg was higher in the COVID-19 pandemic group (29.1%, 15.1%, p<0.001). All patients had their IOP controlled by medications.

Fortunately, no confirmed COVID-19 cases were found among our in-patients or our hospital staff in the COVID-19 pandemic period.

## Discussion

The COVID-19 pandemic has widely impacted patients and healthcare workers. Our study investigated the presenting characteristics and differences in the RRD services, treatment, and surgical outcomes for RRD patients during the COVID-19 pandemic and pre-COVID-19 period. We found a significant reduction of RRD patients during the COVID-19 pandemic. We also found signs of delayed presentation, increased RRD complex in patients, and changes in treatment patterns, but comparable outcomes.

The COVID-19 period took place during Beijing’s top-level emergency COVID-19 response after the spring festival holiday. We chose a pre-COVID-19 period of the same length after the national holiday to exclude the seven-day long holiday effect on the RRD service and its impact on the patients.

### The impact on the patients

Patients with RRD are considered emergency cases and are reported to receive surgery 1–7 days after the onset of symptoms [5, 12–14]. During Beijing’s top-level COVID-19 emergency response, the 14-day quarantine period led to prolonged presurgical waiting times in the COVID-19 pandemic group (28days vs. four days). We investigated the differences between the two groups considering several RRD characteristics related to RRD’s severity. We found a high, equivalent prevalence of PVR in both periods, a higher percentage of CD, and worse presurgical VA in the COVID-19 pandemic.

Prolonged presurgical waiting times are reported to be related to the occurrence of severe PVR [15, 16]. Contrary to the increased PVR prevalence in RRD patients presented during the COVID-19 pandemic lockdown in England and the US [8, 9], we did not find a difference in PVR severity between the COVID-19 pandemic group and the pre-COVID-19 group. The prevalence of PVR in both groups of patients was much higher than previously reported [17–19]. In the pre-COVID-19 period, we, as a tertiary center, received more complicated cases (many of which were referred to our center from other provinces for consultations) with a high prevalence of severe PVR. Due to Beijing’s lockdown during the COVID-19 pandemic, more local patients (data was not shown) were referred to our center, which was the only center providing RRD surgery service. Delays in the presentation may have caused the comparable severity of PVR during the COVID-19 pandemic.

The high prevalence of 4-quadrant RD has contributed to the higher CD prevalence in both groups compared to the level in previous reports, going from 8.6 [20] to 18.79% [21]. This is because the retinal breaks located posterior to the equator may have been related to more severe PVD tractions and more liquation of the vitreous body [14], which may have accelerated the RD progression [22, 23]. The high percentage of patients with retinal breaks located posterior to the equator in the COVID-19 pandemic group may have contributed to the higher RRD-CD percentage (34.2%), which is higher than the percentage for the pre-COVID-19 group (19.6%). The reason for the higher percentage of patients with retinal breaks located posterior to the equator is unknown. Since RRD-CD is related to PVR progression and surgery failure [20, 24], the high percentage of RRD-CD patients makes the RRD cases during the COVID-19 pandemic complicated.

Our findings suggest that the prolonged presurgical waiting times caused by the quarantine period may have aggravated RRD.

### Impact on the medical service

In the early period of the COVID-19 pandemic, there was a shortage of medical protection materials; only one operating room and one group of surgeons were available to carry out RRD surgeries daily. We found a reduction in general anesthesia (48.1%, 83.2%), shortened median length of hospital stay (three hours vs. 35 hours).

### Impact on surgery

PPV and SB are standard procedures for treating RRD. Either could be optimal in some instances [12]. We reported a similar VA (46.8% vs. 40.2% gained more than two lines) and retinal attachment rate (94.9% vs. 94.5%) outcome compared to previously reported [12].

The overall percentage of SB has dropped from 41.4% in the pre-COVID-19 period to 27.8% in the COVID-19 pandemic period. In patients who received SB, the percentage of subretinal drainage has also dropped from 75.5% in the pre-COVID-19 period to 45.4% in the COVID-19 pandemic period.

It has been reported that PPV would be a better choice in the case of RRD patients with a pseudophakic eye [25], severe PVR [17], and CD [26]. The high prevalence of these characteristics in the COVID-19 pandemic group may contribute to the higher prevalence of PPV. Moreover, patients who received SB might need intensive follow-up or second procedures due to postoperative unabsorbing subretinal fluid [27]. It was challenging to arrange intensive follow-ups or short-term second procedures in the COVID-19 pandemic due to reduced outpatients and operation service. It may have an impact on the surgeon’s choice of PPV or SB.

Subretinal fluid drainage may cause subretinal hemorrhage, retinal perforation, vitreoretinal incarceration, eye hypotony, and choroidal detachment [28, 29]. Surgeons in the COVID-19 pandemic tended to give up subretinal fluid drainage during the SB procedure to avoid the complications associated with drainage.

Besides, in the pre-COVID-19 period, there was a group of PM patients with macular hole RD. PM with macular hole RD is common in the Chinese population [30]. Like previously reported, they had some unique characteristics: delayed presentation, slow progression, and worse functional and anatomic prognosis [31], requiring second or combined surgery [28, 30]. Surgery on the PM patient with macular hole RD was suspended during the COVID-19 pandemic because most ophthalmologists and patients preferred follow-up until the visual symptoms were aggregated.

Our results suggested that surgeons in the COVID-19 pandemic were more conservative in their choice of surgical procedures and their patients’ selection.

### Limitations

Since Beijing Tongren Eye Center was the only center in Beijing carrying out RRD surgery services during the COVID-19 pandemic, patients who presented during the COVID-19 pandemic had no other hospital choice. In contrast, patients who presented in the pre-COVID-19 pandemic may have had several other hospitals in Beijing from which to choose. Thus, there was selection bias in the two different periods. The sample sizes were different between the two groups due to the reduction of RRD service in the COVID-19 pandemic. Due to the retrospective study design, the COVID-19 pandemic group’s follow-up period was much shorter than that of the pre-COVID-19 group. There were more patients with silicone oil tamponade in the COVID-19 pandemic group compared to the pre-COVID-19 group when the retinal attachment rate was accessed. The COVID-19 pandemic group patients failed to show whether the retina could be well attached after silicone oil removal. This study was carried out in one tertiary center. We cannot comment on whether the differences found in our study are significant on a national population level. Since the Beijing Tongren eye center was the only center that provided RRD surgery service during the period of the COVID-19 pandemic in Beijing, this study can reflect the changes in RRD service in Beijing.

## Conclusions

In summary, here, we reported a cohort of RRD patients treated during the COVID-19 pandemic. The COVID-19 pandemic has an impact on both patients and ophthalmologists. Compared to those in the pre-COVID-19 period, RRD patients in the COVID-19 pandemic became more challenging due to delayed presentations. Ophthalmologists have to balance the risk of COVID-19 infection during the hospital stay or follow-up and the benefits patients may gain from the surgeries and make more conservative choices of surgical procedures to ensure the surgery’s short-time success.
